# Synergistic effect of two antimicrobial peptides, BP203 and MAP-0403 J-2 with conventional antibiotics against colistin-resistant *Escherichia coli and Klebsiella pneumoniae* clinical isolates

**DOI:** 10.1371/journal.pone.0294287

**Published:** 2023-11-16

**Authors:** Chawalit Chatupheeraphat, Jiratchaya Peamchai, Sirirat Luk-in, Sakda Yainoy, Warawan Eiamphungporn

**Affiliations:** 1 Center for Research Innovation and Biomedical Informatics, Faculty of Medical Technology, Mahidol University, Salaya, Nakhon Pathom, Thailand; 2 Department of Clinical Microbiology and Applied Technology, Faculty of Medical Technology, Mahidol University, Bangkok, Thailand; Abadan University of Medical Sciences, ISLAMIC REPUBLIC OF IRAN

## Abstract

Drug-resistant Enterobacterales infections are a great health concern due to the lack of effective treatments. Consequently, finding novel antimicrobials or combining therapies becomes a crucial approach in addressing this problem. BP203 and MAP-0403 J-2, novel antimicrobial peptides, have exhibited effectiveness against Gram-negative bacteria. In this study, we assessed the *in vitro* antibacterial activity of BP203 and MAP-0403 J-2, along with their synergistic interaction with conventional antibiotics including colistin, rifampicin, chloramphenicol, ceftazidime, meropenem, and ciprofloxacin against colistin-resistant *Escherichia coli* and *Klebsiella pneumoniae* clinical isolates. The minimal inhibitory concentrations (MIC) of BP203 and MAP-0403 J-2 against tested *E*. *coli* isolates were 2–16 and 8–32 μg/mL, respectively. However, for the majority of *K*. *pneumoniae* isolates, the MIC of BP203 and MAP-0403 J-2 were >128 μg/mL. Notably, our results demonstrated a synergistic effect when combining BP203 with rifampicin, meropenem, or chloramphenicol, primarily observed in most *K*. *pneumoniae* isolates. In contrast, no synergism was evident between BP203 and colistin, chloramphenicol, ceftazidime, rifampicin, or ciprofloxacin when tested against all *E*. *coli* isolates. Furthermore, synergistic effects between MAP-0403 J-2 and rifampicin, ceftazidime or colistin were observed against the majority of *E*. *coli* isolates. Similarly, the combined effect of MAP-0403 J-2 with rifampicin or chloramphenicol was synergistic in the majority of *K*. *pneumoniae* isolates. Importantly, these peptides displayed the stability at high temperatures, across a wide range of pH values, in specific serum concentrations and under physiological salt conditions. Both peptides also showed no significant hemolysis and cytotoxicity against mammalian cells. Our findings suggested that BP203 and MAP-0403 J-2 are promising candidates against colistin-resistant *E*. *coli*. Meanwhile, the synergism of these peptides and certain antibiotics could be of great therapeutic value as antimicrobial drugs against infections caused by colistin-resistant *E*. *coli* and *K*. *pneumoniae*.

## Introduction

Antimicrobial resistance poses a global public health threat [1]. In particular, the emergence of multidrug-resistant (MDR) and extensively drug-resistant (XDR) Gram-negative bacteria (GNB) is a significant cause of morbidity and mortality worldwide [2]. Additionally, infections caused by GNB that resist multiple antibiotics bring about substantial economic burdens [3]. Recently, MDR Enterobacterales have been highlighted as potential superbugs, due to their growing antibiotic resistance across various antibiotic classes. The World Health Organization (WHO) listed extended-spectrum β-lactamase (ESBL)-producing and carbapenem-resistant Enterobacterales (CRE) as one of the top priority pathogens responsible for widespread concern [4]. Especially, ESBL-producing Enterobacterales, such as *Escherichia coli* as well as CRE, particularly *Klebsiella pneumoniae* have been increasingly associated with high morbidity rates due to limited treatment options. Currently, only a few antimicrobials, such as colistin, have been used in clinical practice to treat severe infections due to MDR Enterobacterales [5]. Colistin, as an old member of the polymyxin group, has broad-spectrum activity against GNB, including many species of Enterobacterales and is considered the last resort for the treatment of MDR bacterial infections. Unfortunately, the excessive and improper use of colistin has led to the global emergence of colistin-resistant pathogens [6]. Notably, acquired resistance to colistin involving chromosomal and plasmid-mediated resistance has been increasingly detected in several Enterobacterales species [7]. Treating infections caused by colistin-resistant Enterobacterales is challenging due to the limited availability of effective antibiotics.

In recent years, antimicrobial peptides (AMPs) have emerged as promising alternatives for overcoming drug resistance. AMPs possess several advantages over traditional antibiotics, such as a broad-spectrum activity against a wide variety of microbes, rapid bactericidal action, a capability to combat antibiotic-resistant isolates and a low risk of resistance development [8, 9]. Intriguingly, AMPs, in combination with antibiotics, have been demonstrated to enhance antibacterial efficacy, reduce toxic side effects of antimicrobial agents, and minimize selective bacterial resistance [10]. Given these properties, AMPs are proposed as excellent candidates for drug development. Despite their benefits, some drawbacks, such as cytotoxicity and loss activity under physiological conditions, could limit the clinical application of AMPs [11]. In an era marked by a critical shortage of new antibiotics, it is imperative to discover or develop AMPs that overcome these disadvantages for therapeutic use.

In the present study, the *in vitro* antibacterial activity of two novel cationic AMPs, BP203 and MAP-0403 J-2, against colistin-resistant *E*. *coli* and *K*. *pneumoniae* clinical isolates were investigated. BP203 is a BP100 analog featuring a single Lys-to-Arg substitution at position 9. BP100 is a short cationic AMP designed through a combinatorial chemistry approach based on the cecropin A-melittin hybrid [12]. BP203 is composed of 11 amino acids (KKLFKKILRYL-NH_2_) [13]. MAP-0403 J-2 is a peptide analog of MAP-0403, originally designed using an N-terminal fragment of Ixosin-B [14]. Ixosin B is an AMP isolated from the salivary glands of the hard tick *Ixodes sinensis*. MAP-0403 J-2 contains 11 amino acids (KWLRRPWRRWR-NH_2_) [15]. Both peptides have previously shown potent antibacterial activity against a wide array of human pathogens and exhibited little or no hemolytic activity. Nevertheless, there is no study on their *in vitro* antibacterial activities against colistin-resistant Enterobacterales, particularly *E*. *coli* and *K*. *pneumoniae*. Of note, the characteristics of peptides, including thermal and pH stability, as well as stability in serum and under physiological salt conditions, were also evaluated. Additionally, the hemolytic activity and cytotoxicity of these AMPs were investigated. Moreover, the synergistic effects of BP203 and MAP-0403 J-2 in combination with conventional antibiotics comprising colistin, rifampicin, chloramphenicol, ceftazidime, meropenem and ciprofloxacin were studied. To the best of our knowledge, this is the first study to assess the antibacterial activity of these peptides together with the effect of both peptides in combination with conventional antibiotics against colistin-resistant Enterobacterales.

## Materials and methods

### Bacterial isolates, reagents and antibiotics

Ten non-duplicate clinical isolates of colistin-resistant *E*. *coli* (EC) and ten non-duplicate clinical isolates of colistin-resistant *K*. *pneumoniae* (KP) were obtained from the bacterial repository of the Division of Infectious Diseases and Tropical Medicine, Department of Medicine, Faculty of Medicine Siriraj Hospital, Mahidol University. The species identification was conducted through standard biochemical tests. Antimicrobial susceptibility testing was carried out using the disk diffusion method following CLSI guidelines [16]. Additionally, the assessment of colistin resistance in all isolates was performed by broth microdilution following CLSI recommendations. The list of antibiotics used in this study for susceptibility testing was presented in Table 1. According to CLSI breakpoints, colistin resistance was defined as a MIC of ≥4 μg/mL. *E*. *coli* ATCC 25922 and *K*. *pneumoniae* ATCC BAA-1706 (*bla*_KPC_ negative) were used as reference strains throughout the study. The amino acid sequences of BP203 and MAP-0403 J-2 were retrieved from previous studies [13, 15]. These peptides are BP100 and MAP-0403 analogs, respectively. Both peptides were commercially synthesized with C-terminal amidation by GenScript Biotech (Piscataway, NJ, USA) using solid-phase Fmoc chemistry and purified to >90% purity using HPLC. The antibiotics, consisting of chloramphenicol, ceftazidime, meropenem, rifampicin and ciprofloxacin were purchased from Tokyo Chemical Industry (Tokyo, Japan), except for colistin sulfate, which was acquired from Chem-Impex International (Wood Dale, IL, USA). Antibiotic disks and defibrinated sheep blood were obtained from Oxoid (Basingstoke, UK). All culture media were purchased from Becton Dickinson (Frankin Lakes, NJ, USA). All chemicals were obtained from Sigma-Aldrich (St. Louis, MO, USA).

**Table 1:** Antimicrobial susceptibility patterns of all colistin-resistant *E*. *coli* and *K*. *pneumoniae* clinical isolates used in this study.

Isolates	Interpreted results of disk diffusion													COL (μg/mL)
	AMC	CAZ	FEP	CRO	FOX	ETP	ATM	C	CIP	GM	AK	FOS	SXT	
	20/10	30	30	30	30	10	30	30	5	10	30	200	1.25/23.75	
	μg	μg	μg	μg	μg	μg	μg	μg	μg	μg	μg	μg	μg	
EC-01	I	R	R	R	S	S	R	R	R	R	S	S	R	4
EC-02	S	I	R	R	S	S	R	R	I	S	S	S	R	4
EC-03	I	R	R	R	S	S	R	R	S	R	S	S	R	4
EC-04	R	R	R	R	I	S	R	R	R	R	S	R	R	4
EC-05	I	S	SDD	R	S	S	S	R	S	R	S	S	I	4
EC-06	R	R	S	R	S	S	I	S	R	R	S	S	I	4
EC-07	S	I	R	R	S	S	R	R	I	S	S	S	R	8
EC-08	I	S	SDD	R	S	S	S	R	S	R	S	S	I	8
EC-09	S	I	R	R	S	S	R	R	R	R	S	S	R	16
EC-10	S	S	R	R	S	S	R	R	R	R	S	S	R	16
%Resistance	20	40	70	100	0	0	70	90	50	80	0	10	70	100
KP-01	R	R	R	R	R	R	R	R	R	S	S	S	R	4
KP-02	R	R	R	R	R	R	R	R	R	R	R	R	R	8
KP-03	R	R	R	R	R	R	R	S	R	S	R	I	R	32
KP-04	R	R	R	R	R	R	R	S	R	S	R	R	R	16
KP-05	R	R	R	R	R	R	R	S	R	S	I	S	R	16
KP-06	R	R	R	R	R	R	R	R	R	S	R	R	R	16
KP-07	R	R	R	R	R	R	R	I	R	S	S	S	R	64
KP-08	R	R	R	R	R	R	R	S	R	S	S	R	R	128
KP-09	R	R	R	R	R	R	R	I	R	S	S	R	R	32
KP-10	R	R	R	R	R	R	R	R	R	S	S	R	R	128
%Resistance	100	100	100	100	100	100	100	40	100	10	40	60	100	100

^a^ Susceptibility testing for antibiotics except colistin was performed using the disk diffusion method interpreted according to CLSI guideline and abbreviated as follows: R: resistant, I: intermediate, SDD: susceptible-dose dependent, and S: sensitive.

^b^ Colistin susceptibility testing was performed using the broth microdilution method, the MIC ≥4 μg/mL was interpreted as colistin-resistant.

^c^ The abbreviations of antibiotics were designated as follows: AMC, Amoxicillin/clavulanic acid; FOX, cefoxitin; ATM, aztreonam; CAZ, ceftazidime; CRO, ceftriaxone; FEP, cefepime; ETP, ertapenem; C, chloramphenicol; CIP, ciprofloxacin; GM, gentamicin; AK, amikacin; SXT, trimethoprim/sulfamethoxazole; FOS, fosfomycin; COL, colistin (COL).

### Clonal study

The clonal relationship among drug-resistant *E*. *coli* or *K*. *pneumoniae* clinical isolates was analyzed using enterobacterial repetitive intergenic consensus PCR (ERIC-PCR), employing previously described primers: ERIC-1 (5’-ATGTAAGCTCCTGGGGATTCAC-3’) and ERIC-2 (5’-AAGTAAGTGACTGGGGTGAGCG-3’) [17]. Genomic DNA extraction from all isolates was performed using a TIANamp bacteria DNA kit (Tiangen Biotech, Beijing, China) according to the manufacturer’s instructions. The PCR amplification was conducted with a few modifications as follows: initial denaturation at 94°C for 5 min, followed by 35 cycles of denaturation at 94°C for 1 min, annealing at 38°C for 1 min, and extension at 72°C for 3 min, with a final extension at 72°C for 10 min. ERIC-PCR patterns were compared by InfoQuest^TM^FP Software, version 4.5 (Bio-Rad, Hercules, CA, USA) with the Dice coefficient. The dendrogram was constructed by the unweighted pair group method with arithmetic means (UPGMA) using 1.0% optimization and 1.0% band position tolerance. Percentage similarities were used to determine the relatedness of the clones [18].

### Minimum inhibitory concentration (MIC) and minimum bactericidal concentration (MBC)

The MICs of BP203 and MAP-0403 J-2 against clinically colistin-resistant *E*. *coli* and *K*. *pneumoniae* isolates were determined using the broth microdilution method, according to CLSI recommendations [19]. Briefly, the peptides were subjected to serial dilution in cation-adjusted Mueller-Hinton broth (CAMHB) and added to a 96-well microtiter plate (Wuxi NEST Biotechnology, Wuxi, China). Subsequently, bacterial suspensions from freshly mid-log phase cultures were diluted to 10^6^ CFU/mL with CAMHB, and introduced to each well containing the peptides. The final peptide concentrations ranged from 512 to 1 μg/mL. The suspensions were then incubated for 20–24 h at 37°C. The MIC was determined as the lowest peptide concentration with no observable bacterial growth. To investigate the MBC, 10 μL of bacterial suspension that displayed no visible bacterial growth were inoculated on Mueller-Hinton agar (MHA) and incubated for 24 h at 37°C. The MBC was defined as the lowest peptide concentration at which more than 99.9% of the bacterial cells were eradicated.

### Stability testing

The antibacterial activity of BP203 and MAP-0403 J-2 was evaluated concerning their response to temperature, pH, serum, and salt ions using the MIC assay, following the previously mentioned procedure. The MIC values of peptides were determined against *E*. *coli* ATCC29522 and *K*. *pneumoniae* ATCC BAA-1706. In the thermal stability assay, BP203 and MAP-0403 J-2 were individually incubated at 25°C, 37°C, 50°C, 70°C and 90°C for 1 h prior to serial 2-fold dilution and the MIC testing [20]. For the pH stability assay, both peptides were incubated in buffers with different pH values (100 mM buffers: glycine-HCl buffer (pH 2.0), sodium acetate buffer (pH 4.0), sodium phosphate buffer (pH 6.0), Tris-HCl buffer (pH 8.0), and glycine-NaOH buffer (pH 10.0)) for 1 h at 37°C. Then, neutralization was performed before conducting a series of 2-fold dilution and the MIC testing [21]. For serum stability assay, both peptides were exposed to 25% and 50% fetal bovine serum (FBS) for 1 h at 37°C, followed by a heat activation step for 30 min at 60°C. Afterward, the peptide solutions were serially diluted and MIC was subsequently determined [22]. For the salt stability assay, each peptide was treated in MHB supplemented with different salts at their physiological concentrations; NaCl (100, 150, and 200 mM), MgCl_2_ (0.5, 1 and 2 mM), and FeCl_3_ (1, 4 and 8 mM). The serial dilutions of peptides were incubated with tested strains for MIC testing [23]. The MICs were observed after 20–24 h of incubation at 37°C.

### Hemolytic activity assay

Sheep erythrocytes were employed to assess the hemolytic activity of BP203 and MAP-0403 J-2, following previously described with some modifications [24]. Briefly, defibrinated sheep red blood cells (sRBCs) were centrifuged at 1,000× g, 4°C for 10 min. The packed sRBCs underwent three times washes with phosphate buffer saline (PBS), at pH 7.4, and were subsequently suspended in PBS to achieve a concentration of 4% (v/v). BP203 or MAP-0403 J-2 was serially 2-fold diluted, resulting in a concentration ranging from 400 to 12.5 μg/mL. Aliquots of sRBC suspension were mixed with AMP in a 1:1 ratio, yielding final concentrations ranging from 6.25–200 μg/mL. Following an incubation period of 1 h at 37°C, the mixtures were centrifuged at room temperature, 1,000× g for 10 min. The supernatants were collected and transferred to a 96-well microplate. Next, absorbance measurement was taken at 540 nm using a microplate reader (Tecan Group, Zurich, Switzerland). Blank, negative and positive controls were established using sRBCs incubated with PBS alone, BSA solution, and 0.1% Triton X-100, respectively. Additionally, melittin was applied as a positive reference due to its significant hemolytic activity. The percentage of hemolytic activity was calculated with the following equation: Hemolysis (%) = {(Sample absorbance—PBS absorbance)/(0.1% Triton X-100 absorbance—PBS absorbance)} × 100.

### Cytotoxicity assay using MTT

MRC-5 (CCL-171) cell line, a representative of normal lung fibroblast cells, was used to investigate the cytotoxic effects of two AMPs, BP203 and MAP-0403 J-2. The cells were grown in Dulbecco’s Modified Eagle Medium (DMEM, Cytiva Life Sciences, Marlborough, MA, USA), supplemented with 10% fetal bovine serum (FBS), 1% penicillin (10,000 U/mL) and streptomycin (10,000 μg/mL) (Gibco, Waltham, MA, USA). 2×10^4^ cells were seeded into each well of a 96-well plate and allowed to adhere for 24 h. Then, these cells were subjected to exposure to a medium containing a prepared two-fold serial dilution of BP203 or MAP-0403 J-2. The concentration range for this dilution spanned from 200 to 6.25 μg/mL, and this exposure period lasted for an additional 24 h. 3-(4,5-Dimethylthiazol-2-yl)-2,5-Diphenyltetrazolium Bromide (MTT) was introduced into each well to achieve a concentration of 0.5 mg/mL followed by 4 h. incubation. Subsequently, formazan crystals were dissolved through the addition of 100 μL of a solubilizing solution composed of 10% SDS in 0.1 N HCl and the developed color was measured using a microtiter plate reader at an optical density (OD) of 570 nm. Cell viability was calculated as follows: (ODsample/ODcontrol) × 100. Melittin, known for its cytotoxic activity, was used as the positive control. Cytotoxicity was determined by calculating the half-maximal inhibitory concentration (IC_50_), which represents the concentration that reduces cell viability to 50%.

### Checkerboard assay for synergy testing

The synergistic activities of various two-antimicrobial combinations against colistin-resistant *E*. *coli* and *K*. *pneumoniae* isolates were evaluated using the checkerboard assay, which was performed in 96-well plates [25]. Both peptides in combination with different antibiotics including colistin, ceftazidime, meropenem, ciprofloxacin, chloramphenicol and rifampicin were tested against all isolates. Two-fold serial dilutions of peptides and antibiotics were independently prepared in CAMHB. Afterward, a mixture of different concentrations of the antibiotics and peptides was added to the wells of the microplates. Antibiotics and peptides were also dispended alone in the first row and the first column, respectively. Bacterial inoculum was added to the 96-well plate to achieve a final concentration of 5 × 10^5^ CFU/mL. Then, plates were incubated at 37˚C for 20–24 h. MICs were recorded as the minimum concentration of antimicrobial agents without bacterial growth. The interaction between the antimicrobials was determined by calculating the fractional inhibitory concentration index (FICI) according to the formula: FICI = FIC(drug A) + FIC(drug B), where FIC = the MIC of the drug when in the combination/MIC of drug tested individually. FICI index values were interpreted as follows: synergism ≤0.5; no interaction > 0.5–4.0, and antagonism >4.0.

### Time-kill assay for synergy testing

Time-kill assay was conducted according to the method described previously with minor modifications [26]. BP203 was combined with meropenem, chloramphenicol or rifampicin, whereas MAP-0403 J-2 was combined with ceftazidime, chloramphenicol, colistin or rifampicin for testing against selected colistin-resistant *E*. *coli* and *K*. *pneumoniae* isolates. Initially, tubes containing freshly prepared CAMHB supplemented with the sub-inhibitory concentrations of the antimicrobials alone or in combinations were inoculated with tested isolates, achieving a density of 10^6^ CFU/mL in a final volume of 5 mL and incubated at 37°C. Aliquots were withdrawn at time intervals of 0, 1, 2, 4, 6, 8 and 24 h post-inoculation, and serially diluted in saline for determination of viable counts. Diluted samples (50 μL) were plated on MHA plates, and bacterial counts were determined after 18 h of incubation at 37°C. The bactericidal activity was defined as ≥3 log10 CFU/mL reduction in the colony count, relative to the initial inoculum. Synergism and indifference were defined as a ≥2 log10 and <2 log10 decrease in the CFU count, respectively when the combination was compared with the most active single drug after 24 h of incubation [27].

### Statistical analysis

All experiments were carried out in two independent assays, which each experiment performed in triplicate. When the results differed for MIC or MBC determinations, a third test was performed. Time-kill experiments were performed in duplicate. Shapiro-Wilk test was used to analyze the normal distribution (P value <0.05). Upon confirming no significant deviation from normality, one-way analysis of variance (ANOVA) was employed to analyze the significance of differences among groups. All statistical calculations were performed using GraphPad Prism version 8.4.3. Values of P <0.05 were considered statistically significant.

## Results

### Antimicrobial susceptibility testing

The antimicrobial susceptibility of ten clinical isolates of *E*. *coli* and ten clinical isolates of *K*. *pneumoniae* were determined. The percentage of antibiotic resistance is shown in Table 1. For *E*. *coli*, all isolates exhibited resistance to most third- and fourth-generation cephalosporins, they showed susceptibility to ertapenem (ETP). Additionally, the majority of them demonstrated resistance to aztreonam (ATM), chloramphenicol (C), ciprofloxacin (CIP), gentamicin (GM) and trimethoprim/sulfamethoxazole (SXT). Regarding *K*. *pneumoniae*, all isolates exhibited a resistance pattern against third- and fourth-generation cephalosporins. In addition, all isolates were also resistant to ertapenem (ETP) and deemed as carbapenem-resistant *K*. *pneumoniae*. These isolates also displayed resistance to amoxicillin/clavulanic acid (AMC), cefoxitin (FOX), aztreonam (ATM), ciprofloxacin (CIP) and trimethoprim/sulfamethoxazole (SXT). Remarkably, all *E*. *coli* and *K*. *pneumoniae* isolates were resistant to colistin (COL) with MICs ≥4 μg/mL. Overall, our results demonstrated that all *E*. *coli* and *K*. *pneumoniae* isolates showed MDR phenotypes and some *K*. *pneumoniae* isolates exhibited possibly XDR phenotypes.

### Clonal study

**Fig 1:**
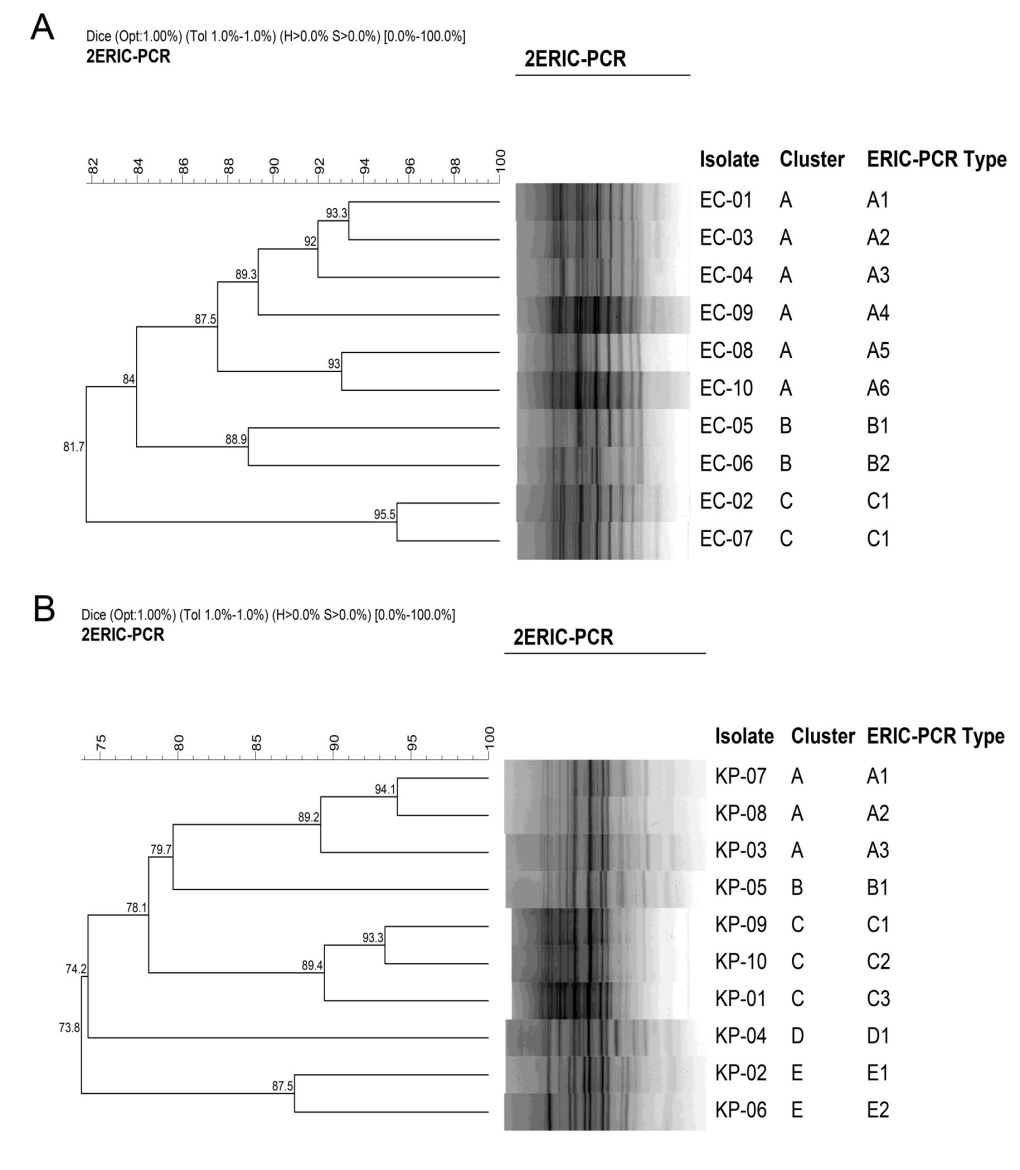
Dendrogram of genetic similarity generated by ERIC-PCR among clinically colistin-resistant isolates. (A) The cluster and ERIC-PCR types of ten isolates of *E*. *coli* (B) The cluster and ERIC-PCR types of ten isolates of *K*. *pneumoniae*.

The clonal diversity of ten *E*. *coli* and ten *K*. *pneumoniae* clinically colistin-resistant isolates was determined. Among the group of ten clinical *E*. *coli* isolates, a division into three distinct clusters labeled A to C emerged, alongside the identification of nine unique ERIC-PCR types. This differentiation was established using a cut-off of 85% and 95% genetic similarity, respectively (Fig 1A). Notably, within Cluster C, an ERIC-PCR type denoted as C1 displayed two closely correlated patterns. It is of significance that two isolates, specifically EC-02 and EC-07, exhibited similar antibiotic patterns, indicating a probable shared clonal origin. Among ten *K*. *pneumoniae* clinical isolates, a classification into five clusters designated A to E was achieved, with the concurrent distinction of ten diverse ERIC-PCR types using the similar cut-off as mentioned above (Fig 1B). All isolates demonstrated different antibiotic susceptibility profiles. As a result, all *K*. *pneumoniae* isolates were considered clonally unrelated strains, and none of the identical ERIC-PCR patterns were found among these isolates.

### MIC and MBC determination

The MIC and MBC values of BP203 and MAP-0403 J-2, evaluated against colistin-resistant *E*. *coli* and *K*. *pneumoniae* isolates, are presented in Table 2. The MICs of BP203 and MAP-0403 J-2 tested against colistin-resistant *E*. *coli* clinical isolates were 2–16 and 8–64 μg/mL, respectively. Furthermore, BP203 inhibited the growth of colistin-resistant *K*. *pneumoniae* clinical isolates at concentrations of 16–512 μg/mL, while MAP-0403 J-2 displayed weak or no antibacterial activity against colistin-resistant *K*. *pneumoniae* (MIC ≥512 μg/mL). Of particular note is the observation that both peptides displayed elevated MIC and MBC values for colistin-resistant *K*. *pneumoniae* isolates when compared with those of a reference strain. Additionally, most MIC and MBC values showed either equivalence or a variance not exceeding a single dilution.

**Table 2:** The MIC and MBC values of BP203 and MAP-0403 J-2 against *E*. *coli* and *K*. *pneumoniae* reference strains and colistin-resistant *E*. *coli* and *K*. *pneumoniae* clinical isolates.

Organisms	BP203		MAP-0403 J-2	
	MIC (μg/mL)	MBC (μg/mL)	MIC (μg/mL)	MBC (μg/mL)
***E*. *coli* ATCC 25922**	4	4	16	16
**EC-01**	2	2	16	32
**EC-02**	8	8	32	32
**EC-03**	2	2	8	16
**EC-04**	2	4	8	8
**EC-05**	8	8	32	64
**EC-06**	2	2	32	32
**EC-07**	8	8	32	32
**EC-08**	16	16	32	64
**EC-09**	2	2	8	32
**EC-10**	8	8	32	64
***K*. *pneumoniae* ATCC BAA-1706**	4	4	16	16
**KP-01**	16	16	>512	>512
**KP-02**	256	256	128	256
**KP-03**	128	128	>512	>512
**KP-04**	512	512	>512	>512
**KP-05**	32	32	256	512
**KP-06**	256	256	>512	>512
**KP-07**	128	128	>512	>512
**KP-08**	256	256	>512	>512
**KP-09**	512	512	>512	>512
**KP-10**	512	512	>512	>512

### Thermal and pH stability

The thermal and pH stability of BP203 and MAP-0403 J-2 were investigated by determining the MIC values after exposing the peptides to various temperatures and pH buffers for 1 h. Notably, both peptides retained their antibacterial activity against *E*. *coli* ATCC 25922 and *K*. *pneumoniae* ATCC BAA-1706 when exposed to temperatures of 50°C, 70°C, or 90°C, indicating their good stability at high temperatures (Table 3). In addition, these two peptides also demonstrated consistent antibacterial activity across a wide pH spectrum, ranging from 2.0 to 10.0 (Table 4). These results suggested that both peptides exhibited great thermal- and pH-resistant stability.

**Table 3:** The MIC values of BP203 and MAP-0403 J-2 against *E*. *coli* and *K*. *pneumoniae* reference strains after different temperature treatments.

Bacterial Strains	Temperature (°C)	Antimicrobial activity MIC (μg/mL)	
		BP203	MAP-0403 J-2
*E*. *coli* ATCC 25922	25 (control)	4	16
	37	4	16
	50	4	16
	70	4	16
	90	4	16
*K*. *pneumoniae* ATCC BAA-1706	25 (control)	4	16
	37	4	16
	50	4	16
	70	4	16
	90	4	16

**Table 4:** The MIC values of the BP203 and MAP-0403 J-2 against *E*. *coli* and *K*. *pneumoniae* reference strains after different pH treatments.

Bacterial Strains	pH	Antimicrobial activity MIC (μg/mL)	
		BP203	MAP-0403 J-2
*E*. *coli* ATCC 25922	7 (control)	4	16
	2	4	16
	4	4	16
	6	4	16
	8	4	16
	10	4	16
*K*. *pneumoniae* ATCC BAA-1706	7 (control)	4	16
	2	4	16
	4	4	16
	6	4	16
	8	4	16
	10	4	16

### Salt and serum stability

The stability of BP203 and MAP-0403 J-2 in the presence of salt and serum was also investigated through the determination of the MIC values after exposure to different physiological salts and varying concentrations of FBS for 1 h. The antibacterial activity of both AMPs remained largely unaltered following exposure to 25% and 50% FBS (Table 5). Moreover, the results revealed that treatment with various concentrations of NaCl, MgCl_2_, and FeCl_3_ had no negligible effects on the antibacterial activity of these peptides against tested strains (Table 6). Altogether, these findings indicated that both peptides possess good resistance to serum as well as physiological salts, such as NaCl, MgCl_2_, and FeCl_3_.

**Table 5:** The MIC values of the BP203 and MAP-0403 J-2 against *E*. *coli* and *K*. *pneumoniae* reference strains after FBS treatments.

Bacterial Strain	%FBS	Antimicrobial activity MIC (μg/mL)	
		BP203	MAP-0403 J-2
*E*. *coli* ATCC 25922	0 (control)	4	16
	25	4	16
	50	8	32
*K*. *pneumoniae* ATCC BAA-1706	0 (control)	4	16
	25	4	16
	50	4	16

**Table 6:** The MIC values of the BP203 and MAP-0403 J-2 against *E*. *coli* and *K*. *pneumoniae* reference strains in the presence of physiological salts.

Bacterial Strain	Salt	Conc.	Antimicrobial activity MIC (μg/mL)	
			BP203	MAP-0403 J-2
*E*. *coli* ATCC 25922	Control	0	4	16
	NaCl	100 mM	8	32
		150 mM	16	64
		200 mM	16	64
	MgCl_2_	0.5 mM	4	16
		1 mM	4	16
		2 mM	8	16
	FeCl_3_	1 μM	4	16
		4 μM	4	16
		8 μM	4	16
*K*. *pneumoniae* ATCC BAA-1706	Control	0	4	16
	NaCl	100 mM	8	32
		150 mM	16	64
		200 mM	16	64
	MgCl_2_	0.5 mM	4	16
		1 mM	8	16
		2 mM	8	16
	FeCl_3_	1 μM	4	16
		4 μM	8	16
		8 μM	8	32

### Cytotoxic activity effects of BP203 and MAP-0403 J-2 against mammalian cells

**Fig 2:**
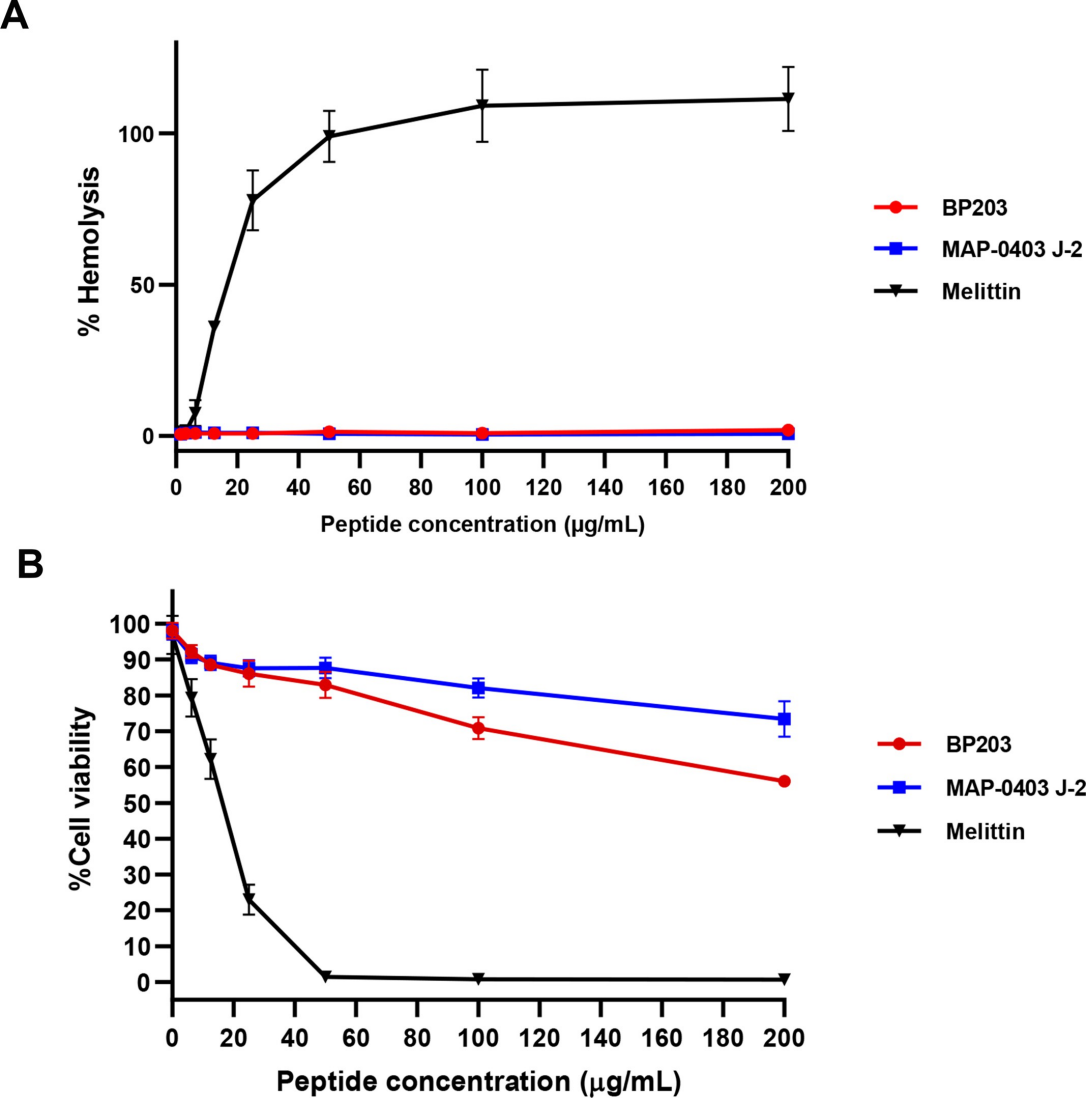
Cytotoxic activity effects of BP203 and MAP-0403 J-2 against mammalian cells. (A) The hemolytic activity of BP203 and MAP-0403 J-2 against sheep red blood cell. (B) The evaluation of cytotoxicity of BP203 and MAP-0403 J-2 against human lung fibroblast cell line, MRC-5, using MTT assay. Melittin was used as a positive reference of cytotoxic peptide.

The potential mammalian cytotoxicity was assessed using two assays: hemolytic activity and cytotoxicity via the MTT assay. As depicted in Fig 2A, melittin, a peptide known for its cytotoxic activity, exhibited a high level of hemolytic activity with a 50% hemolysis (HL50) at 15 μg/mL. Remarkably, both peptides demonstrated negligible hemolysis even at concentrations as high as 200 μg/mL, which is approximately 50 and 12.5 times the MICs against *E*. *coli* and *K*. *pneumoniae* reference strains, respectively, correlating with the cytotoxicity assay results. BP203 and MAP-0403 J-2 demonstrated minimal cytotoxicity, reducing cell viability to 56.01% and 73.43%, respectively, at their highest concentration. Significantly, the IC_50_ values for both peptides were >200 μg/mL. Notably, MAP-0403 J-2 was less cytotoxic than BP203. In contrast, melittin, a highly toxic peptide, remarkably reduced cell viability to approximately 1% at a concentration of 50 μg/mL, with an IC_50_ of 16.13 μg/mL, as shown in Fig 2B.

### *In vitro* evaluation of synergy by checkerboard assay

The synergistic effects resulting from combining each peptide with common antibiotics were evaluated as the FICI. The results of FICI of BP203 and MAP-0403 J-2 in combination with six antibiotics against colistin-resistant *E*. *coli* and *K*. *pneumoniae* clinical isolates are provided in Tables 7 and 8, respectively. All combinations of BP203 with antibiotics, except rifampicin, showed no synergistic effect on all colistin-resistant *E*. *coli* isolates. It is worth noting that the BP203-rifampicin combination displayed a synergistic effect of only 20% (2/10). For colistin-resistant *K*. *pneumoniae* isolates, the most significant synergistic effect was observed with the BP203-meropenem and BP203-rifampicin combinations, both at 90% (9/10), This was followed by BP203-chloramphenicol combination at 70% (7/10), BP203-ceftazidime combination at 30% (3/10), and BP203-ciprofloxacin combination at 10% (1/10). Notably, BP203-colistin had no synergistic effect on all *K*. *pneumoniae* isolates. Meanwhile, the combination of MAP-0403 J-2-rifampicin demonstrated the greatest potential synergistic effect on *E*. *coli* isolates with a synergistic effect of 90% (9/10), followed by MAP-0403 J-2-ceftazidime at 80% (8/10), MAP-0403 J-2-colistin at 70% (7/10), and MAP-0403 J-2-chloramphenicol at 50% (5/10). However, MAP-0403 J-2-ciprofloxacin was ineffective on *E*. *coli* isolates. Intriguingly, the combination of MAP-0403 J-2-rifampicin also showed the highest synergistic effect at 90% (9/10) on colistin-resistant *K*. *pneumoniae* isolates. MAP-0403 J-2-chloramphenicol had a synergistic effect of 60% (6/10), followed by MAP-0403 J-2-ceftazidime at 50% (5/10), and MAP-0403 J-2-ciprofloxacin at 10% (1/10) against *K*. *pneumoniae* isolates. The combination of MAP-0403 J-2-meropenem and MAP-0403 J-2-colistin showed no synergistic effect against all *K*. *pneumoniae* isolates. It is important to highlight that no antagonism was detected in any combination of each peptide and antibiotic tested across all the screened *E*. *coli* and *K*. *pneumoniae* isolates.

**Table 7:** FICIs of BP203 combined with conventional antibiotics to treat clinically isolated colistin-resistant *E*. *coli* and *K*. *pneumoniae*.

Isolates	FICI					
	BP203					
	COL	MEM	C	RIF	CIP	CAZ
EC-01	0.625	ND	0.5078	1	1.0156	1
EC-02	1	ND	1	0.3125	0.75	0.75
EC-03	0.75	ND	1	1.25	1.0078	0.75
EC-04	0.75	ND	0.5313	1.25	1.0039	0.75
EC-05	0.75	ND	1	0.75	1	0.75
EC-06	2	ND	0.75	1.25	1.0156	1.0313
EC-07	0.625	ND	0.75	0.75	1.125	0.625
EC-08	2	ND	1	0.75	1	1
EC-09	0.625	ND	0.75	1	1.0156	1
EC-10	0.625	ND	0.625	0.5	0.5078	1
**Synergism**	**0 (0%)**	**ND**	**0 (0%)**	**2 (20%)**	**0 (0%)**	**0 (0%)**
**No interaction**	**10 (100%)**	**ND**	**10 (100%)**	**8 (80%)**	**10 (100%)**	**10 (100%)**
KP-01	1.0156	0.5	0.75	0.156	0.375	0.56
KP-02	1.0078	0.375	0.266	0.023	1	0.156
KP-03	1.0019	0.5	0.375	0.1875	0.5625	0.75
KP-04	1.0039	0.375	0.5156	0.0625	0.5039	1.007
KP-05	1.0625	0.75	0.5625	0.56	1.0313	0.5078
KP-06	1.0078	0.2813	0.375	0.078	0.5039	0.0781
KP-07	2.0078	0.5	0.265	0.187	0.5031	0.625
KP-08	1.0019	0.375	0.375	0.3125	0.5039	0.625
KP-09	0.5039	0.375	0.1406	0.0775	1.0078	0.75
KP-10	0.502	0.2578	0.1406	0.156	1.0078	0.1875
**Synergism**	**0 (0%)**	**9 (90%)**	**7 (70%)**	**9 (90%)**	**1 (10%)**	**3 (30%)**
**No interaction**	**10 (100%)**	**1 (10%)**	**3 (30%)**	**1 (10%)**	**9 (90%)**	**7 (70%)**

^a^ green shading indicates synergy interaction.

^b^ ND; not done.

^c^ The abbreviations of antibiotics were indicated as follows: COL, colistin; MEM, meropenem; C, chloramphenicol; RIF, rifampicin; CIP, ciprofloxacin; CAZ, ceftazidime.

**Table 8:** FICIs of MAP-0403 J-2 combined with conventional antibiotics to treat clinically isolated colistin-resistant *E*. *coli* and *K*.*pneumoniae*.

Isolates	FICI					
	MAP-0403 J-2					
	COL	MEM	C	RIF	CIP	CAZ
EC-01	0.625	ND	0.75	0.25	1	0.3125
EC-02	0.5	ND	0.75	0.375	1	0.5
EC-03	0.375	ND	0.5	0.5313	0.75	0.3125
EC-04	0.75	ND	0.5	0.5	0.75	0.5
EC-05	0.375	ND	1	0.375	1	0.3125
EC-06	0.5625	ND	0.75	0.25	1	1.25
EC-07	0.5	ND	1.5	0.3125	1	0.625
EC-08	0.5	ND	0.5	0.2813	0.75	0.375
EC-09	0.3125	ND	0.5	0.1875	1	0.25
EC-10	0.5	ND	0.5	0.1875	0.625	0.375
**Synergy**	**7 (70%)**	**ND**	**5 (50%)**	**9 (90%)**	**0 (0%)**	**8 (80%)**
**No interaction**	**3 (30%)**	**ND**	**5 (50%)**	**1 (10%)**	**10 (100%)**	**2 (20%)**
KP-01	1.002	0.75	0.251	0.0703	1.0005	0.1270
KP-02	1.0039	2	0.3125	0.125	0.375	0.1875
KP-03	1.0039	1.0078	0.75	0.0781	1.0005	0.625
KP-04	1.002	1.5	0.375	0.1563	0.5010	0.75
KP-05	1.002	1.25	0.3125	0.0781	1.0005	0.625
KP-06	1.0625	1	0.2505	0.375	0.5625	0.375
KP-07	2.002	1.0156	0.5005	0.125	1.0005	0.5
KP-08	1.002	1.5	0.501	0.1406	1.0005	0.625
KP-09	1.0039	1	0.501	0.75	1	0.625
KP-10	1	0.625	0.3125	0.0938	1.0005	0.375
**Synergy**	**0 (0%)**	**0 (0%)**	**6 (60%)**	**9 (90%)**	**1 (10%)**	**5 (50%)**
**No interaction**	**10 (100%)**	**10 (100%)**	**4 (40%)**	**1 (10%)**	**9 (90%)**	**5 (50%)**

^a^ green shading indicates synergy interaction.

^b^ ND; not done.

^c^ The abbreviations of antibiotics were indicated as follows: COL, colistin; MEM, meropenem; C, chloramphenicol; RIF, rifampicin; CIP, ciprofloxacin; CAZ, ceftazidime.

### Time-kill assay

**Fig 3:**
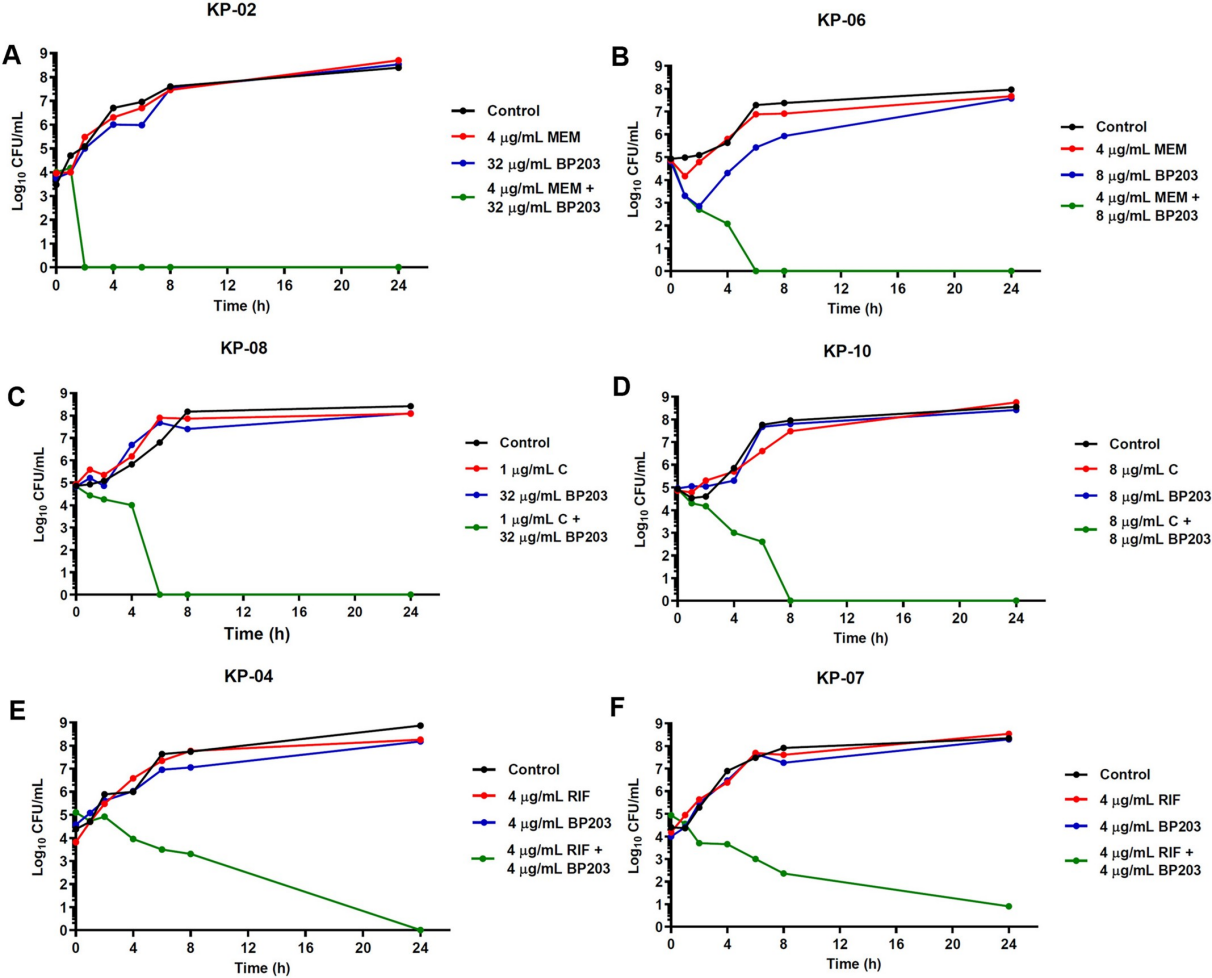
Time-kill curves of synergistic combinations of BP203 with conventional antibiotics against colistin-resistant *K*. *pneumoniae* isolates. (A, B) the combination of BP203 with meropenem. (C, D) the combination of BP203 with chloramphenicol. (E, F) the combination of BP203 with rifampicin.

**Fig 4:**
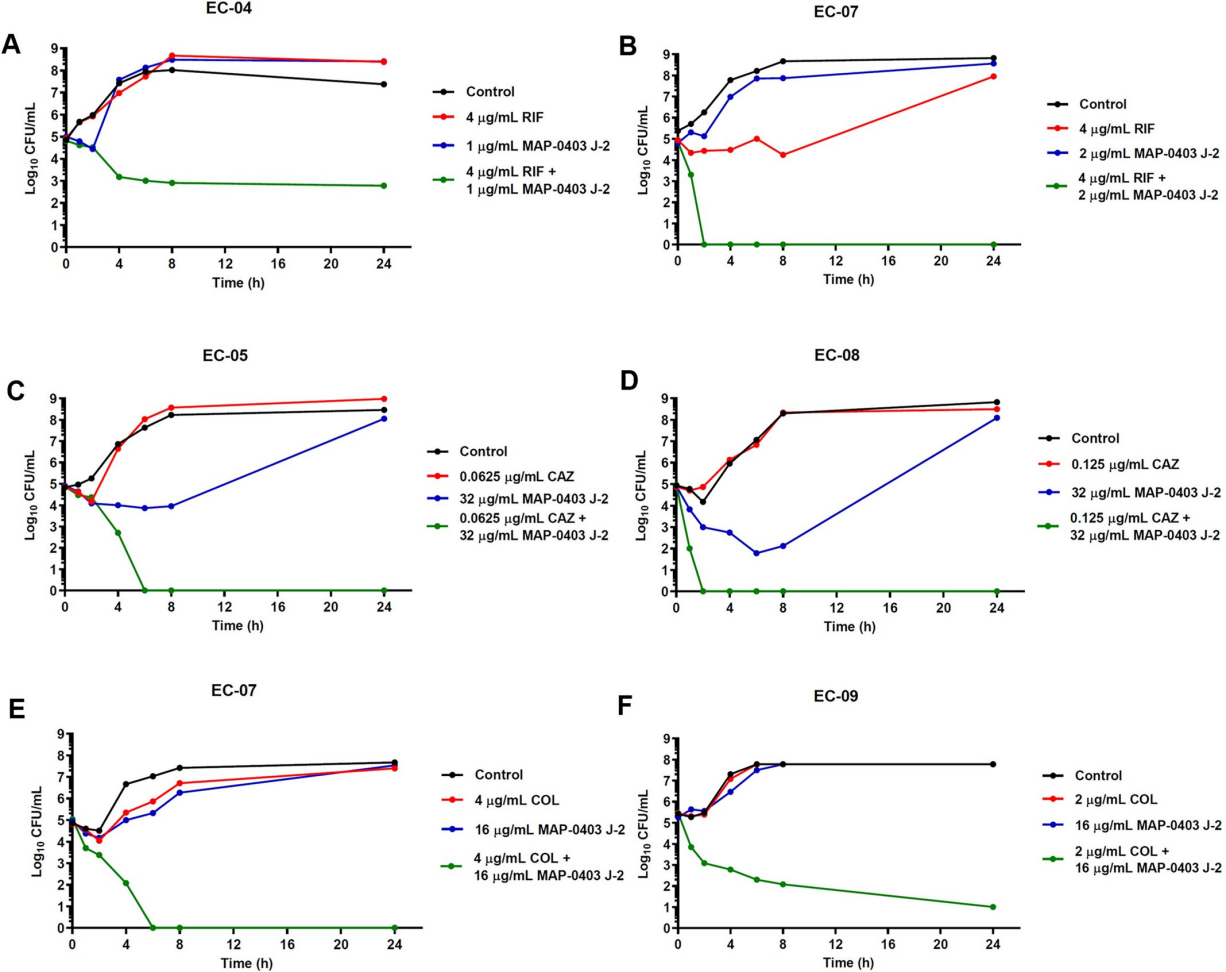
Time-kill curves of synergistic combinations of MAP-0403 J-2 with conventional antibiotics against colistin-resistant *E*. *coli* isolates. (A, B) The combinations of MAP-0403 J-2 with rifampicin. (C, D) The combinations of MAP-0403 J-2 with ceftazidime. (E, F) The combinations of MAP-0403 J-2 with colistin.

**Fig 5:**
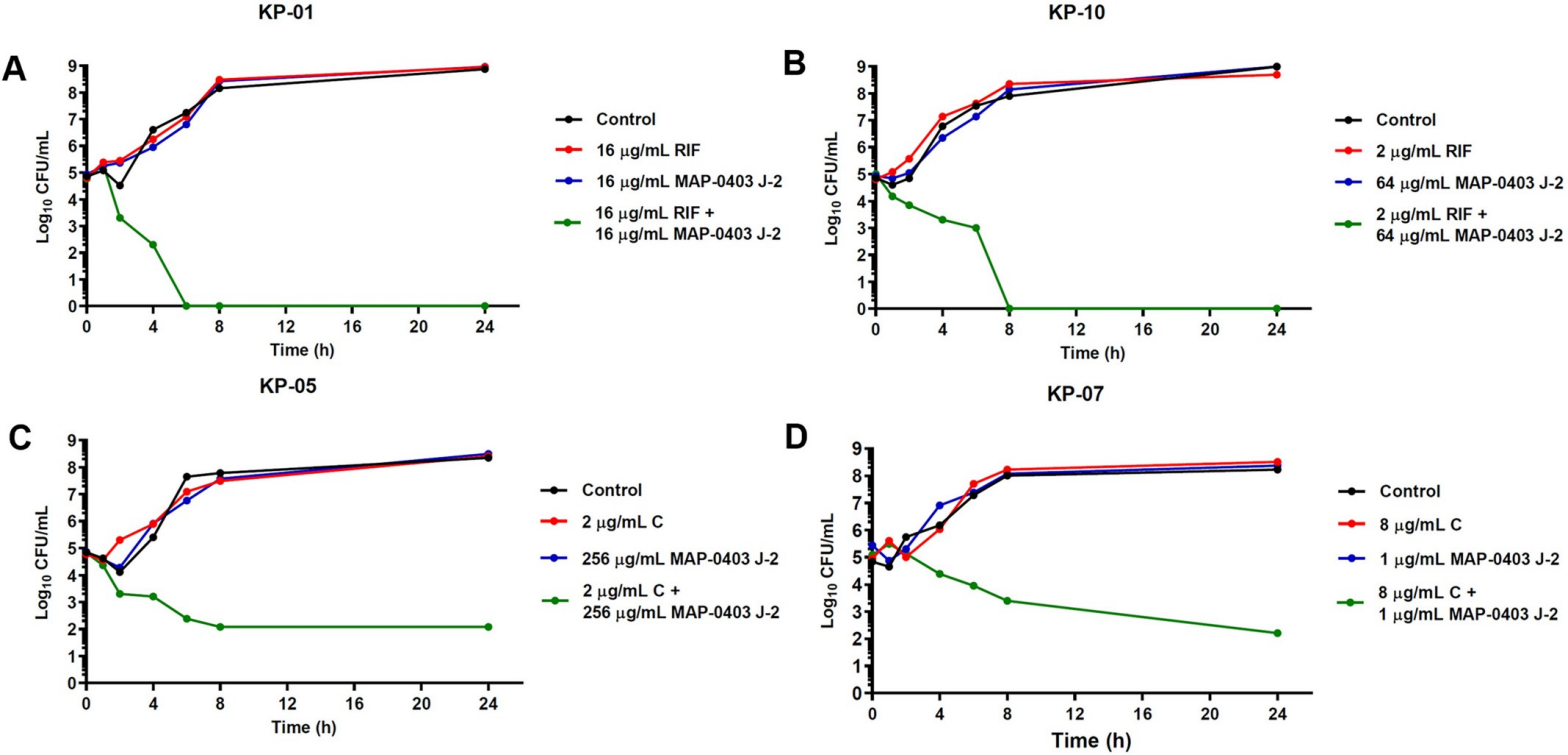
Time-kill curves of synergistic combinations of MAP-0403 J-2 with conventional antibiotics against colistin-resistant *K*. *pneumoniae* isolates. (A, B) The combinations of MAP-0403 J-2 with rifampicin. (C, D) The combinations of MAP-0403 J-2 with chloramphenicol.

Time-kill assay was performed to confirm the synergistic results that were obtained from the checkerboard assay. Five and eight randomly selected *E*. *coli* and *K*. *pneumoniae* isolates were tested, respectively. The peptide and drug concentrations used for the time-kill curves were derived from checkerboard results, with FICI <0.5. Time-kill curves against all tested *E*. *coli* and *K*. *pneumoniae* isolates are represented in Figs 3–5. Mostly, bacteria exposed to each peptide or drug alone displayed a killing curve comparable to the control culture not exposed to antimicrobial agents. As shown in Fig 3, the combination of BP203 and antibiotics (meropenem, rifampicin, and chloramphenicol) resulted in more rapid killing, and all *K*. *pneumoniae* isolates treated with these combinations demonstrated a dramatic decrease in viable cells >2 log10 (CFU/mL)-fold by 24 h compared to that observed with either agent alone. Additionally, all combination treatments effectively suppressed bacterial growth, reducing the number of viable bacterial cells by >3 log10 CFU/mL over 24 h. These findings thus confirmed the bactericidal activity of BP203 when combined with meropenem, rifampicin, or chloramphenicol. The results also exhibited that the combination of MAP-0403 J-2 and antibiotics (rifampicin, ceftazidime and colistin) against tested *E*. *coli* isolates along with the combination of MAP-0403 J-2 and antibiotics (rifampicin and chloramphenicol) against tested *K*. *pneumoniae* isolates caused a significant reduction in viable cells >2 log10 (CFU/mL)-fold by 24 h compared to that observed with either agent alone (Figs 4 and 5). Moreover, all combinations noticeably inhibited bacterial growth (>3 log10 decrease in colony counts) over 24 h. In summary, the combination of MAP-0403 J-2 and each antibiotic showed good synergistic and bactericidal activity against all tested isolates within 24 h. These results clearly demonstrated the synergistic potential of the successful peptide-antibiotic combinations in killing the representative bacterial strains that were employed in the study.

## Discussion

The escalating rise of bacterial antibiotic resistance threatens global health and gives a driving force to the search for new antimicrobial agents as alternatives. AMPs have gained significant attention due to their rapid bactericidal action, antimicrobial activity in the micromolar range and multifaceted mechanism of action [28]. Nevertheless, certain AMP’s exhibit reduced antimicrobial potency in certain environments, such as high temperatures and acid-base circumstances [29]. Additionally, many AMPs perform sub-optimally function in the presence of physiological salt concentrations and are prone to degradation in serum [30]. Consequently, the finding of short AMPs possessing both high antimicrobial activity, thermal and pH stability as well as stability under physiological conditions is a desirable objective. In this study, the *in vitro* antibacterial activity of two AMPs, BP203 and MAP-0403 J-2, against colistin-resistant *E*. *coli* and *K*. *pneumoniae* clinical isolates was explored. All studied *E*. *coli* isolates showed MDR phenotypes, and likewise, all screened *K*. *pneumoniae* isolates exhibited MDR phenotypes, with some even demonstrating extensively drug-resistant (XDR) phenotypes. According to ERIC-PCR patterns, all colistin-resistant *E*. *coli* and *K*. *pneumoniae* isolates were clonally unrelated strains, except EC-02 and EC-07, which represent identical ERIC-PCR patterns. This highlights the pronounced diversity among the investigated colistin-resistant isolates, rendering them valuable subjects for evaluating the antibacterial potential of BP203 and MAP-0403 J-2. A previous study showed that BP203 possessed high antibacterial activity against reference strains of *E*. *coli* and *K*. *pneumoniae* with both MICs 8.5 μg/mL [13]. Moreover, MAP-0403 J-2 also exhibited potent antibacterial activity against *E*. *coli* with MIC 2.5 μM (~4.2 μg/mL) [15]. These results are in good agreement with our results, in which the MICs of BP203 against *E*. *coli* and *K*. *pneumoniae* reference strains were 4 μg/mL. However, the MIC of MAP-0403 J-2 against *E*. *coli* ATCC 29522 was slightly higher than in a previous study (16 μg/mL). Interestingly, our results suggested that both AMPs were equally active against colistin-susceptible and -resistant strains of *E*. *coli*. However, the greater MICs of both AMPs against colistin-resistant *K*. *pneumoniae* were observed. It is consistent with a previous study, showing that the colistin-resistant *A*. *baumannii* were less susceptible to BP203 than their parental strains [13]. Of note, the MICs of MAP-0403 J-2 against most colistin-resistant *K*. *pneumoniae* isolates were >512 μg/mL, indicating that this peptide had no or low antibacterial activity against these resistant strains. The higher MICs of both AMPs against colistin-resistant *K*. *pneumoniae* could be possibly cross-resistance between colistin and these AMPs since all tested *K*. *pneumoniae* strains were colistin-resistant. Contrarily, the colistin resistance in *E*. *coli* did not confer cross-resistance with tested AMPs. Some previous studies reported the cross-resistance between colistin and other AMPs due to the fact that colistin and AMPs share the same mechanism of action [31–33]. Nevertheless, some studies also showed contrasting results [34, 35]. Our results implied that the cross-resistance between colistin and AMPs may vary depending on bacterial species. Altogether, our findings indicated that BP203 and MAP-0403 J-2 seemed to be more potent against colistin-resistant *E*. *coli* rather than colistin-resistant *K*. *pneumoniae*.

The stability of AMPs is crucial for storage and applications [36]. Temperature and pH are critical parameters impacting AMP stability. In addition, the good stability of AMPs to physiological salts and serum has been considered a desirable property for clinical applications [37]. In this study, BP203 and MAP-0403 J-2 retained antibacterial activity under elevated temperatures, various pH conditions, and in the presence of physiological salts and serum environment. Previous studies revealed that both AMPs had low hemolytic activity. The hemolytic activity of BP203 at 150 μM (~300 μg/mL) was 31%, while the hemolytic activity of MAP-0403 J-2 at 100 μM (~678 μg/mL) was 3.4% [13, 15]. Of note, the reported hemolytic activity of both AMPs was relatively consistent with our study, in which these AMPs exhibited no hemolytic activity at 200 μg/mL. To the best of our knowledge, the cytotoxicity of BP203 and MAP-0403 against mammalian cells has not been previously reported. This is the first study to evaluate the cytotoxicity of BP203 and MAP-0403 J-2 against mammalian cell lines. Our results displayed that both AMPs exhibited slight toxicity toward MRC-5, human fetal lung fibroblast cells with IC_50_ >200 μg/mL. As expected, they have significantly lower cytotoxicity than melittin. Accordingly, our findings suggested that BP203 and MAP-0403 J-2 had great stability without significant hemolytic activity and cytotoxicity, which might probably be promising candidates for treating colistin-resistant *E*. *coli* infections.

Combination therapy involving AMPs and common antibiotics provides an effective therapeutic option to improve the efficiency of bacterial killing, reduce the therapeutic dose, prevent the occurrence of resistance and reduce side effects [38]. Given that most AMPs target membranes, perturbing their structures, the combinatory therapy AMP-antibiotic arises as an efficient tool to increase antibiotic bioavailability [39]. Recently, combination therapy has gained prominence in combating MDR bacterial infections. In the literature, the synergistic effects of AMPs and antibiotics have been reported to be effective against a range of resistant strains of bacteria [40–43]. Using checkerboard assay, BP203 and MAP-0403 J-2 exhibited the best synergistic effects with rifampicin against colistin-resistant *E*. *coli* and *K*. *pneumoniae* isolates. The exact mechanism underlying the synergism between both AMPs and rifampicin is still unclear, although it is anticipated that AMPs improved cell membrane permeability may elevate intracellular rifampicin accumulation within GNB, bolstering its effectiveness [44]. Intriguingly, rifampicin has been recently introduced in combination therapy, particularly with colistin against MDR GNB [45–47]. Moreover, several studies previously reported the effectively combined therapy between AMPs and rifampicin against MDR Enterobacterales [48–50]. However, clinical evidence to support the use of combined therapeutic regimens with rifampicin in the treatment of MDR Enterobacterales infections is still lacking and requires to be evaluated. Therefore, this combination should be considered only when there are no other reasonable options [51]. In our study, most combinations of BP203 with antibiotics showed no synergism against colistin-resistant *E*. *coli* isolates, whereas this peptide exhibited good synergistic effects with meropenem and chloramphenicol against colistin-resistant *K*. *pneumoniae*. MAP-0403 J-2 displayed notable synergistic effects with ceftazidime, colistin, and chloramphenicol against colistin-resistant *E*. *coli* isolates, along with exhibiting good synergism with chloramphenicol, and ceftazidime against colistin-resistant *K*. *pneumoniae*. It is worth noting that peptides displayed a strikingly synergistic interaction with meropenem or ceftazidime. This discovery is particularly advantageous in clinical applications since these antibiotics are frequently employed to treat Enterobacterales infections and have minimal adverse effects. The mechanisms contributing to synergism between both AMPs and various antibiotics are still unknown and should be further investigated. However, one possible mechanism for the synergistic effects between AMPs and chloramphenicol is the disruption of the bacterial membrane, facilitating the delivery of chloramphenicol into cells, where the drug can act on intracellular targets. The synergism between AMPs and chloramphenicol against drug-resistant Enterobacterales was previously reported [52, 53]. Additionally, the synergism between AMP and β-lactams may be implicated in the combined effects of both AMPs and β-lactams on the cell envelope, associated with the disruption of the membrane by AMPs and the inhibition of cell wall biosynthesis by β-lactams [50]. Interestingly, MAP-0403 J-2 acts synergistically with colistin against most colistin-resistant *E*. *coli* isolates, but BP203 could not synergize with colistin against all *E*. *coli* isolates. This finding implied that the specific binding mechanism to bacterial membrane likely varies for different AMPs; therefore, modification of lipid A and other membrane moieties might not universally confer resistance to all AMPs [54]. In the case of MAP-0403 J-2, it might contribute to potentiating the membrane perturbing activity of colistin, leading to microbial death at lower concentrations than those needed by the single agent. The synergistic effects of some AMPs and colistin against colistin-resistant *E*. *coli* have been elucidated in previously described [54–56]. Of note, our results also suggested that the synergistic effects of each AMP with different antibiotics could depend on bacterial species. Bacterial specificity of AMPs that are acting synergistically has been previously described [53, 57]. This is probably one of the limitations for clinical application since a broad antimicrobial activity of AMP used for single or combination therapy will be preferable to extend the treatments among clinically relevant GNB.

The synergistic activity of both AMPs with conventional antibiotics was verified using a time-kill assay, a method serving for a more dynamic assessment of bactericidal and combinatorial effects. The time-kill assay provides more precise data regarding the effect of the combinations since the measurements are taken over time, detecting bactericidal activities and bacterial regrowth [58]. Moreover, this method is able to detect the synergistic effects for some isolates that were not detected by checkerboard assay. Noteworthy, our data showed a good agreement between time-kill and checkerboard results. Interestingly, BP203-meropenem and BP203-chloramphenicol combinations completely eradicated the growth of colistin-resistant *K*. *pneumoniae* within 4 h and 8 h, respectively. Meanwhile, the results showed that the bactericidal action (≥3 log10 CFU/mL) of BP203 in combination with rifampicin was achieved after 6 h. For colistin-resistant *E*. *coli*, MAP-0403 J-2 exerted the synergistic effect when combined with rifampicin, ceftazidime or colistin. Although the growth pattern of two representatives tested against each drug was different depending on the strains, all combinations exhibited the bactericidal effect. Moreover, complete elimination of colistin-resistant *K*. *pneumoniae* growth was also observed within 4–8 h when MAP-0403 J-2 was combined with rifampicin. The bactericidal effect was also detected when MAP-0403 J-2 was combined with chloramphenicol. Of note, no regrowth was observed in all combinations. According to these combination results, it seems to be promising to combine both AMPs and these antibiotics where antibacterial agents provide a bactericidal effect.

In conclusion, our results showed that BP203 and MAP-0403 J-2 possess potent antibacterial activity against colistin-resistant *E*. *coli* and may serve for the treatment of this drug-resistant pathogen. These AMPs also exhibited high thermal and wide pH stability, alongside great stability in the presence of serum and physiological salts. Moreover, both AMPs displayed low *in vitro* hemolytic activity and cytotoxicity. Intriguingly, combination therapy with both AMPs, particularly against colistin-resistant *K*. *pneumoniae*, demonstrates a promising and potent therapeutic avenue addressing “superbug” infections. Our findings encourage further exploration of possible applications of these synergistic combinations in the treatment of colistin-resistant Enterobacterales infections. *In vivo* experiments and clinical settings remain to be achieved for a deeper understanding of the therapeutic efficacy, safety, and tolerability of these synergistic interactions.

## Supporting information

S1 File The checkerboard results of BP203 and MAP-0403 J-2, in combination with conventional antibiotics against *E*. *coli* and *K*. *pneumoniae* clinical isolates. (XLSX)

S2 File The results of the cytotoxic and hemolytic activities of BP203 and MAP-0403 J-2. (XLSX)
